# Electroacupuncture on Trigeminal Nerve-Innervated Acupoints Ameliorates Poststroke Cognitive Impairment in Rats with Middle Cerebral Artery Occlusion: Involvement of Neuroprotection and Synaptic Plasticity

**DOI:** 10.1155/2020/8818328

**Published:** 2020-09-07

**Authors:** Yu Zheng, Zongshi Qin, Bun Tsoi, Jiangang Shen, Zhang-Jin Zhang

**Affiliations:** ^1^School of Chinese Medicine, LKS Faculty of Medicine, The University of Hong Kong, Hong Kong, China; ^2^Department of Chinese Medicine, The University of Hong Kong Shenzhen Hospital (HKU-SZH), Shenzhen, Guangdong 518053, China

## Abstract

Poststroke cognitive impairment (PSCI) is a severe sequela of stroke. There are no effective therapeutic options for it. In this study, we evaluated whether electroacupuncture (EA) on the trigeminal nerve-innervated acupoints could alleviate PSCI and identified the mechanisms in an animal model. The male Sprague-Dawley rat middle cerebral artery occlusion (MCAO) model was used in our study. EA was conducted on the two scalp acupoints, *EX-HN3* (*Yintang*) and *GV20* (*Baihui*), innervated by the trigeminal nerve, for 14 sessions, daily. Morris water maze and novel object recognition were used to evaluate the animal's cognitive performance. Neuroprotection and synaptic plasticity biomarkers were analyzed in brain tissues. Ischemia-reperfusion (I/R) injury significantly impaired spatial and cognition memory, while EA obviously reversed cognitive deterioration to the control level in the two cognitive paradigms. Moreover, EA reversed the I/R injury-induced decrease of brain-derived neurotrophic factor, tyrosine kinase B, N-methyl-D-aspartic acid receptor 1, *α*-amino-3-hydroxy-5-methyl-4-isoxazole propionic acid receptor, *γ*-aminobutyric acid type A receptors, Ca^2+^/calmodulin-dependent protein kinase II, neuronal nuclei, and postsynaptic density protein 95 expression in the prefrontal cortex and hippocampus. These results suggest that EA on the trigeminal nerve-innervated acupoints is an effective therapy for PSCI, in association with mediating neuroprotection and synaptic plasticity in related brain regions in the MCAO rat model.

## 1. Introduction

Stroke is the second leading cause of disability-related death worldwide [1]. Increasing numbers of stroke patients suffer from poststroke cognitive impairment (PSCI), characterized by poor performance on abstract thinking, memory, orientation, and similar functions. However, the mechanism of PSCI has not been fully elucidated. Thus, the therapeutic options for it are limited. Some commonly used drugs in Alzheimer's disease (AD) have shown positive effects in PSCI patients, such as cholinesterase inhibitors in which one of them is donepezil [2]. But the benefits in global and daily cognitive function are inconsistent, making it difficult to assess the efficacy [3].

In cerebral ischemia, multiple pathophysiological changes can be observed, including brain edema, neuronal loss, and changes in synaptic plasticity [4]. Most synapses are found in the dendrites, which are the primary determinants of neuronal integration and information processing [5]. The primary activities of the neurotrophic factor are improving synaptic transfer, promoting synaptic plasticity, developing synaptogenesis, and neuroprotection, which could ameliorate synaptic dysfunction [6]. Synaptic plasticity can also be altered by changing the quantity of synaptic neurotransmitter receptors [7], such as N-methyl-D-aspartate receptor (NMDAR), *α*-amino-3-hydroxy-5-methyl-4-isoxazole propionic acid receptor (AMPAR), and *γ*-aminobutyric acid type A receptor (GABA_A_R) which are associated with neuronal development, synaptic plasticity, learning, and memory [8, 9]. Note that NMDAR is activated by Ca^2+^ influx [10]. Therefore, the intracellular Ca^2+^/calmodulin-dependent protein kinase II (CaMKII) is also a critical molecular determinant of neuroprotection and cognitive function [11].

The hippocampus, one of the most intensely studied areas of the brain, is assumed to be involved in spatial memory [12]. In addition to spatial memory, nonspatial recognition memory is the ability to distinguish whether something is familiar or not. Evidence from human neuroimaging has indicated that the prefrontal cortex (PFC) contributes to recognition memory [13, 14]. Both the hippocampus and PFC have a strong association with cognitive function in different kinds of memories, which have been chosen to be the related brain regions in our study.

In the past decades, complementary medicine has become popular in clinical services and one of them is acupuncture. Acupuncture is gradually gaining popularity for the treatment of neurological diseases, including vascular dementia [15], vascular cognitive impairment no dementia [16], PSCI [17], and stroke [18]. However, more evidence is needed to evaluate its clinical effects. We have explored a novel acupuncture regimen named electroacupuncture trigeminal nerve stimulation (EA/TNS) [19]. Recently, we have published one directly related clinical trials of EA/TNS on PSCI patients, suggesting that EA on forehead acupoints could reduce cognitive deterioration of stroke patients [20]. We want to explore the mechanism of it through this study.

Therefore, we hypothesize that acupuncture could reverse PSCI in animal models by neuroprotection and regulating synaptic plasticity in the hippocampus and PFC. One classical PSCI animal model (middle cerebral artery occlusion (MCAO)) was used in our study. Corresponding behavior tests were conducted to evaluate cognitive performance. Biomarkers for neuroprotective activity and synaptic plasticity-related proteins were tested.

## 2. Materials and Methods

### 2.1. Animals and Experimental Procedure

**Figure 1:**
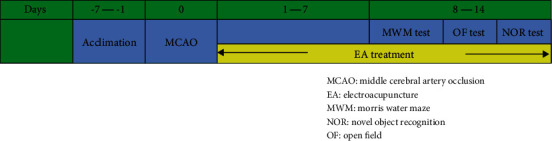
The experimental timeline.

The experiment protocol was approved by the Committee on the Use of Live Animals in Teaching and Research (CULATR: 4840-18) of LKS Faculty of Medicine of the University of Hong Kong. The male Sprague-Dawley rats weighing 270 ± 20 g were housed three to four per cage and maintained on a 12 h light/dark cycle at 23°C with water and food available ad libitum. Animals that were dying before the experimental endpoint were replaced to ensure *n* = 10 each group for quantification according to some previous studies [21, 22]. Body weight was monitored during the whole experiment. The whole experimental procedure was conducted in the lab animal unit (LAU) of the University of Hong Kong, and the animals were monitored by a veterinarian every day to assess their welfare. The whole experimental timeline is shown in Figure 1.

### 2.2. MCAO Procedure

The MCAO operation was established according to the previous protocol [23]. Briefly, rats were anaesthetized with 4% isoflurane (Abbott, IL, USA) and maintained at 2% isoflurane via inhalation. Rats were then placed on a warm pat to maintain body temperature. Under an operating microscope, after creating a 2 cm width incision in the neck, the common carotid artery (CCA) was located under the muscles. The CCA, internal carotid artery (ICA), and external carotid artery (ECA) were separated and ligated with a 6/0 nylon thread (Ningbo Medical Needle Co. Ltd., Ningbo, China) under an operating microscope. A nylon thread with a diameter of 0.36 mm with a silicone tip (L3600, Jialing Co. Ltd., Guangzhou, China) was inserted into the ICA from a stump on the ECA. The thread bolt was set into the bifurcation point of the left middle cerebral artery (MCA), inducing the blockage of blood flow. The CCA was transiently ligated during these processes. After 2 hours of occlusion, the bolt was taken out such that the CCA was unobstructed to allow blood reperfusion to the ischemic area. The muscle layer was sutured with a 5/0 polyglactin suture, and the layers were incised with a 3/0 nylon suture. Sham-operated rats were subjected to the same procedure as above without suture insertion into the MCA. All rats were kept on the warm pad until they awoke when they were returned to their cages. Liquid food was provided if needed. Intraoperative monitoring (IOM) forms were required during the operation.

### 2.3. Treatment

After 24 h recovery from the operation, in the EA group, acupuncture needles (0.25 × 15 mm, MOCM, China) were inserted tangentially to the skin into the acupoints, *EX-HN3* (*Yintang*) and *GV20* (*Baihui*) to a depth of 10 mm. Electroacupuncture on these two acupoints is the most commonly used regimen in the acupuncture treatment of various psychiatric disorders [24]. The needle handles were taped onto the surface at the two acupoints. The rats could move freely in the box during the treatment (Supplementary File 1). The needles were connected to the output terminals of the electroacupuncture instrument (ITO Physiotherapy & Rehabilitation Co., Tokyo, Japan) with continuous-wave stimulation at a frequency of 2 Hz, intensity of 1 mA, pulse width of 100 *μ*s for 10 min, for 14 days. The determination of the low frequency is used because it could exert broader modulatory effects on central neurochemical systems compared to that of the high frequency and has been widely used in clinical practice and animal studies [25]. The same wire stabilization without acupuncture needle procedure was used during the treatment, to minimize potential confounders in the model and sham-operated groups. As such, all three groups of animals experienced similar handling-induced stress. All EA treatment was conducted in the morning.

### 2.4. Behavioral Test

The Morris water maze and novel object recognition tests were performed to evaluate rats' spatial learning and memory and cognition memory abilities, respectively. The open field test was used in this research to evaluate the anxiety level and locomotor level. All testing was conducted in the morning, videotaped, and analyzed with video tracking software (EthoVision, Noldus, Netherlands).

#### 2.4.1. Morris Water Maze Test

The water maze test was conducted in a circular diameter of a 150 cm tank with a height of 50 cm water. Four different markers, equally spaced along the circumference of the tank, were pasted on the tank to divide it into 4 quadrants. Subsequently, water at 24 ± 2°C was poured into the water maze. The tank was positioned in a well-lit testing room. The test was conducted for six consecutive days within two parts, training and probe testing. On the first day, a transparent cylinder platform was above the water level so that the rat could find that there exist a platform in the tank and posited in one quadrant. The rat in each group facing the wall was lowered gently into the water for free swimming for 60 seconds twice, respectively, and randomly selected from the other three quadrants. On the second to the fifth day, the platform was submerged and hidden from the rat's view but still in the same quadrant as the first day. The similar layout and training method would be employed. If the rat could find and rest on the platform within 60 seconds, the time would be recorded. If the rat could not find the platform within 60 seconds, it would be guided to the platform for 10 seconds, and the time would be recorded as 60 seconds. The average time was recorded in these two training.

The probe test was carried out one day after the last training trial. The platform was removed, and the rat would be placed in a new start position for freely swimming 60 seconds. The duration spent in the targeted quadrant in which the arena platform was located and the frequency into the targeted quadrant was recorded.

After each day swimming test, the rats would be dried and put under an infrared lamp to keep them warm.

#### 2.4.2. Novel Object Recognition Test

The apparatus is a 100 × 100 × 60 cm Plexiglas arena with black walls and floor. During the training phase, the rat has encountered two equal sample objects (cube with water, made of plastic) in opposite corners for 10 min. 24 hours later, the rat was returned to the same apparatus and presented with a familiar object and a novel object (cylinder with water, made of plastic) for 10 min. Exploration is defined as exploring the object at a distance ≤ 2 cm or touching with its nose. The total exploring time for objects was calculated, and the discrimination index was calculated and analyzed through the following formula [26]:

(1) $$\begin{array}{c} \text{Index}=\frac{\left(\text{Time spent exploring novel object}-\text{time spent exploring unchanged object}\right)}{\left(\text{Time spent exploring novel object}+\text{time spent exploring unchanged object}\right)}. \end{array}$$

#### 2.4.3. Open Field Test

The open field apparatus is a 100 × 100 × 60 cm Plexiglas arena with black walls and floor with a 30 × 30 cm central zone. Testing followed in a dim-light condition without the presence of experimenters. The total duration time between the central and surrounding zones was recorded in a 10 min test period. The apparatus was cleaned with 75% alcohol between tests.

### 2.5. Tissue Preparation and Sections

The rats were transcranially perfused with PBS under anesthesia to collect brain tissue for further examination after the above behavior tests. The hippocampus and PFC were extracted with radioimmunoprecipitation assay (RIPA) buffer (Sigma-Aldrich, USA) supplemented with 1% protein inhibitor phenylmethanesulfonylfluoride (PMSF, Sigma-Aldrich, USA). After centrifugation, the supernatants were collected for Western blot.

### 2.6. Western Blot Analysis

The mentioned supernatants were analyzed according to the standard Western blot protocol. Equal amounts of proteins were separated by 10% SDS-PAGE gel, transferred onto polyvinylidene difluoride membranes (PVDF, 0.22 *μ*M, Bio-Rad Laboratories, Inc.), and blocked with 5% BSA blocking buffer. Immunodetection was performed with primary antibodies against PSD-95 (1 : 2000, Abcam), NeuN, AMPAR (1 : 2000, Cell Signaling Technology), *β*-actin (1 : 5000, Cell Signaling Technology), CaMKII, BDNF, GABA_A_R, NMDAR1, and TrkB (1 : 2000, Santa Cruz Biotechnology, Inc.) at 4°C overnight. This was followed by coincubation with related secondary antibodies for 2 h at 4°C. Bands were detected by enhanced chemiluminescence staining (GE Healthcare, IL, USA). The images were captured by Gel-Doc System (Bio-Rad, Laboratories, Inc.). The intensity of protein bands was quantified by scanning densitometry with Image Lab 5.1 software (Bio-Rad, Laboratories, Inc.).

### 2.7. Immunofluorescence

After phosphate-buffered saline perfusion under anesthesia, the rat was continuously perfused with 4% paraformaldehyde (PFA), and the whole brain was removed and fixed in 4% PFA for at least one week. Then, the brains were immersed with 30% sucrose solution for dehydration until the tissue reached the bottom of the tube totally at 4°C. The fixed and dehydrated brain was mounted with OCT compound (Leica, Germany), and coronal brain sections (30 *μ*m thick) were cut using a microtome cryostat (Leica, Germany) at −20°C. The cerebral slices were blocked for 1 hour and incubated at 4°C overnight in the following primary antibodies: mouse anti-NeuN (1 : 200, Millipore) and rabbit anti-PSD-95 (1 : 200, Cell signaling technology). The slices were then incubated with fluorescent-dye-conjugated secondary antibodies (DyLight 594-conjugated goat anti-mouse, 1 : 200, Abcam; DyLight 488-conjugated goat anti-rabbit, 1 : 200, Invitrogen) for 1 h at room temperature. The slides were stained with 4′,6-diamidino-2-phenylindole (DAPI) for 15 min. Images were captured using a Zeiss confocal microscope (Zeiss, LSM 780, Germany).

### 2.8. Statistical Analysis

Values were expressed as the mean ± standard error of the mean and subjected to one-way analysis of variance (ANOVA) followed by Dunnett's multiple comparison test for multiple comparisons to detect a between-group statistical difference in behavioral variables (duration in the water maze test, novel object recognition test, and open field test), using Western blot analysis. Two-way ANOVA was used for the body weight during the 5-day training latency data of water maze. The group, time point, and the interaction between the group and time point were treated as fixed effects. GraphPad Prism 7.0 software was used for the statistical analysis. Statistical significance was defined as *p* < 0.05 with a two-sided test.

## 3. Results

### 3.1. Body Weight Change during the Experiment

**Figure 2:**
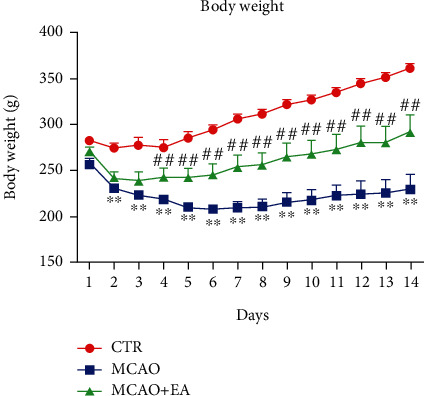
Body weight change during the experiment. Data are expressed as mean ± SEM, where ^∗∗^*p* < 0.01 compared to the control group and ^##^*p* < 0.01 compared to the model group (*n* = 10 each group). CTR: control; MCAO: middle cerebral artery occlusion; EA: electroacupuncture.

Firstly, we investigated the effect of EA on the recovery of I/R injury rats, especially on the body weight. The MCAO operation induced body weight loss (Figure 2). During the whole treatment period, a significant between-group difference (*F*_2,378_ = 258.7, *p* < 0.0001), not only to the time effect (F_13,378_ = 7.54, *p* < 0.0001) but also to the interaction effect between group and time, is detected (*F*_26,378_ = 2.534, *p* < 0.0001), while the body weight increased significantly in the EA group compared to the model group during most of the treatment days.

### 3.2. Cognitive Performance in the Water Maze

**Figure 3:**
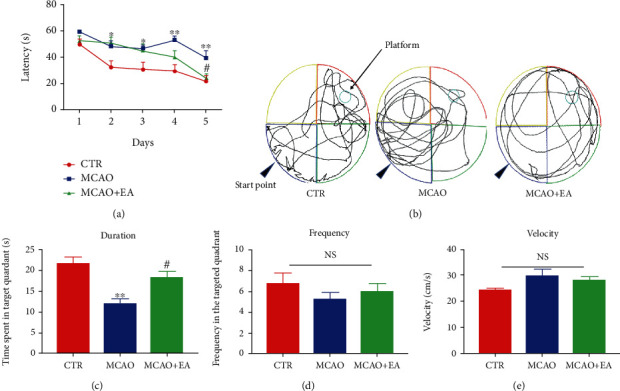
Effects of the MCAO and EA in the water maze test. Escape latency to the invisible platform in the training phase (a), representative swimming track (b), time spent in targeted quadrant (c), frequency in the targeted quadrant (d), and velocity in the water maze (e). Data are expressed as mean ± SEM, where ^∗^*p* < 0.05 and ^∗∗^*p* < 0.01 compared to the control group and ^#^*p* < 0.05 compared to the MCAO group (*n* = 10 each group). NS: no significance; CTR: control; MCAO: middle cerebral artery occlusion; EA: electroacupuncture.

Then, we explored one of the main effect indicators, spatial learning and memory ability, of rat by the Morris water maze test. During the 5-day training latency data of the water maze, two-way ANOVA revealed that there is a significant between-group difference (*F*_2,135_ = 19.07,*p* < 0.0001), in either time effect (*F*_4,135_ = 13.89, *p* < 0.0001). From training day 2 to day 4, a significant difference between the model and control group has been found (*p*_day2_ = 0.0175, *p*_day3_ = 0.0171, and *p*_day4_ = 0.0003), but no difference between the model and EA treatment group (*p*_day2_ = 0.8896, *p*_day3_ = 0.9579, and *p*_day4_ = 0.059). At day 5, multiple comparisons further showed that significantly longer latency was found in the comparison between the model group and the control group (*p* = 0.0068) and the EA treatment group (*p* = 0.025). No interaction effect between group and time is detected (*F*_8,135_ = 1.18, *p* = 0.3157). Significant effects were revealed on the duration spent in the targeted quadrant among these groups after one-way ANOVA (*F*_2,27_ = 11.69, *p* = 0.0002). Dunnett's multiple comparisons indicated that operation decreased the time in the quadrant compared to the control group (*p* = 0.0001), while EA could apparently increase it compared to the MCAO group. But there is no significant difference in frequency in the targeted quadrant (*F*_2,27_ = 0.8151, *p* = 0.4532) and velocity (*F*_2,27_ = 3.195, *p* = 0.0568) in the probe test (*p* = 0.0087) (Figure 3). The results above indicate that there is a decline in the spatial learning and memory in the MCAO group and could be reversed by EA treatment.

### 3.3. Cognitive Performance in Novel Object Recognition

**Figure 4:**
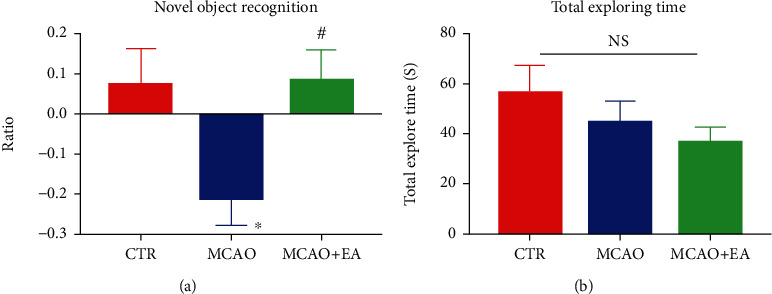
Effects of the MCAO and EA in the novel object recognition test. The discrimination index of the novel object (a). Negative ratio means the rats preferred to explore the familiar object rather than the novel object. Total exploring time in test phases (b). Data are expressed as mean ± SEM, where ^∗^*p* < 0.05, compared to the control group, and ^#^*p* < 0.05 compared to the MCAO group (*n* = 10 each group). NS: no significance; CTR: control; MCAO: middle cerebral artery occlusion; EA: electroacupuncture.

Unlike orientation in the Morris water maze test, the novel object recognition test uses the innate animal preference for novelty, if they could recognize the novel object compared to the familiar objects [26]. This function is dominated by the PFC rather than by the hippocampus in the water maze test [27]. In the novel object recognition test, one-way ANOVA indicated significant effects in the discrimination index (*F*_2,27_ = 4.388, *p* = 0.0224). The discrimination index of the MCAO group was negative, which means the rats in this group preferred to explore the familiar object than the control (*p* = 0.0332), and EA could increase this index compared to the MCAO group (*p* = 0.0273). These results indicate that the recognition function is affected in the MCAO group, and EA could ameliorate this dysfunction. However, there is no difference in the total exploring time (*F*_2,27_ = 1.218, *p* = 0.3115) (Figure 4), indicating that there is no neophobia among these groups.

### 3.4. Anxiety Level in the Open Field Test

**Figure 5:**
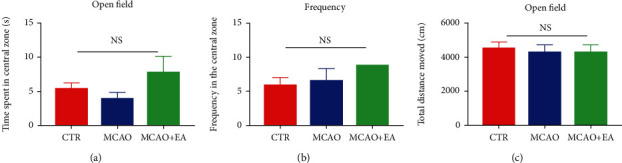
Effects of the MCAO and EA in the open field test. Time spent in the central zone (a), frequency in the central zone (b), and total distance moved (c). Data are expressed as mean ± SEM (*n* = 10 each group). NS: no significance; CTR: control; MCAO: middle cerebral artery occlusion; EA: electroacupuncture.

It has been indicated that anxiety could disrupt cognitive function [28]. Therefore, we determined whether these operations, including the MCAO surgical operation and EA treatment, would influence the rats' anxiety. The open field test is a well-known behavioral test to assess rodents' anxiety [29]. Typically, rodents prefer to stay near the walls rather than the central region while exploring the box, a behavior called thigmotaxis. Spending less time in the periphery is a sign of anxiolysis [30]. Results showed that no significant differences were found in the time spent (*F*_2,27_ = 1.578, *p* = 0.2248) and frequency in the central zone (*F*_2,27_ = 0.8632, *p* = 0.4331) and total distance travelled (*F*_2,27_ = 0.09577, *p* = 0.9090) in the open field test (Figure 5), indicating that the surgery and EA treatment affect neither anxiety level nor locomotor function.

### 3.5. The Expression of Biomarkers for Neuroprotective Activity and Functional Neuroplasticity in Brain Tissues

Summarizing the above behavioral test results, EA treatment could ameliorate the cognitive function. Molecular mechanism would be explored to understand the underlying mechanisms. There are 6 related biomarker expressions detected in the hippocampus and PFC with Western blot. These biomarkers were the proteins on the BDNF/TrkB signaling pathway as well as neurotransmitter receptors and its downstream protein.

**Figure 6:**
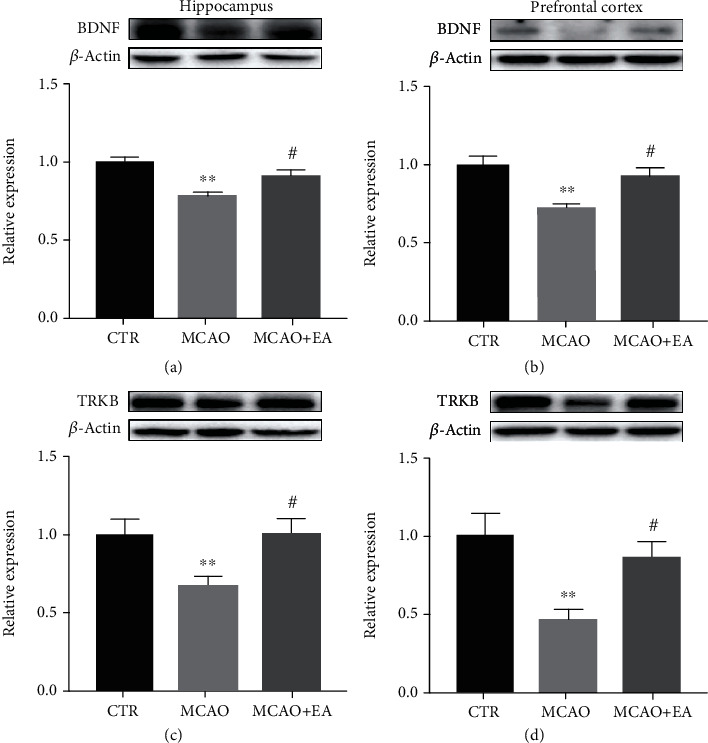
Effects of the MCAO and EA in neuroprotective-related proteins. BDNF (a, b) and TrkB (c, d) expressions in the hippocampus and PFC. Value is expressed as mean ± SEM, where ^∗^*p* < 0.05 and ^∗∗^*p* < 0.01 compared to control group and ^#^*p* < 0.05 compared to the MCAO group (*n* ≥ 5 each group). CTR: control; MCAO: middle cerebral artery occlusion; EA: electroacupuncture.

One-way ANOVA revealed marked effects on the BDNF/TrkB signaling pathway in the hippocampus (*F*_2,21_ ≥ 4.134, *p* ≤ 0.0306) and in the PFC (*F*_2,15_ ≥ 5.901, *p* ≤ 0.0129) (Figure 6). The MCAO surgery significantly downregulated the expression of these two proteins in both brain regions than the control group (*p* < 0.0421). However, the EA treatment could reverse these biomarker levels almost back to the control group level (*p* < 0.0461).

**Figure 7:**
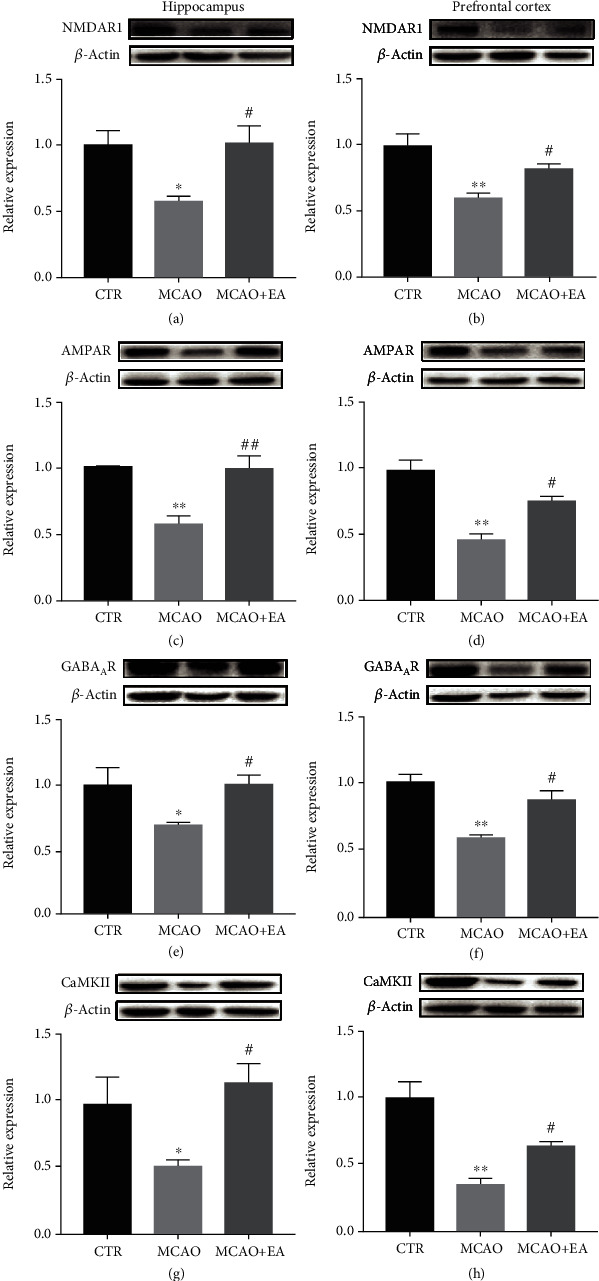
Effects of the MCAO and EA in neurotransmitter receptor proteins. NMDAR1 (a, b), AMPAR (c, d), GABA_A_R (e, f), and CaMKII (g, h) in the hippocampus and PFC, measured using Western blot analysis. Value is expressed as mean ± SEM, where ^∗^*p* < 0.05 and ^∗∗^*p* < 0.01 compared to the control group and ^#^*p* < 0.05 and ^##^*p* < 0.01 compared to the MCAO group (*n* ≥ 3 each group). CTR: control; MCAO: middle cerebral artery occlusion; EA: electroacupuncture.

Meanwhile, significant effects could be found on the expression of functional neuroplasticity biomarkers, NMDAR1, AMPAR, GABA_A_R, and CaMKII, in the hippocampus (*F*_2,12_ ≥ 4.138, *p* ≤ 0.0387) and PFC (*F*_2,12_ ≥ 10.53, *p* ≤ 0.038) (Figure 7). The MCAO operation markedly decreased the expression of these proteins in the brain regions (*p* < 0.0478). Furthermore, EA significantly reversed these expressions to the control level (*p* < 0.0483). These results show that stroke could downregulate the neurotrophin signaling pathway as well as the expression of neurotransmitter receptors. However, EA could reverse these biomarkers.

### 3.6. The Expression of the Structural Synaptic Plasticity-Related Protein in Brain Tissues

**Figure 8:**
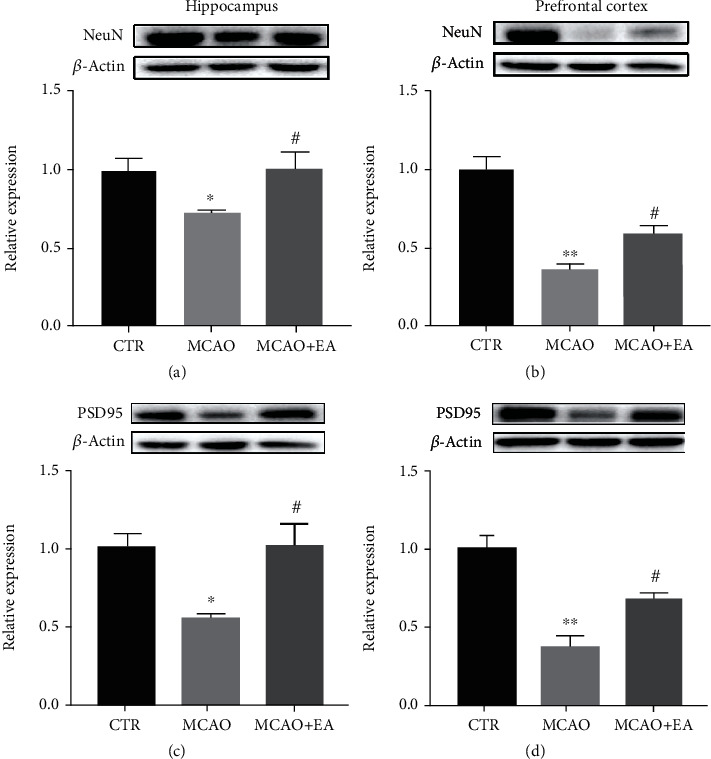
Effects of the MCAO and EA in synaptic-related proteins. Effects of MCAO and EA on the expression of the neuroplasticity biomarkers, NeuN (a, b) and PSD-95 (c, d) in the hippocampus and PFC. Value is expressed as mean ± SEM, where ^∗^*p* < 0.05 and ^∗∗^*p* < 0.01 compared to the control group and ^#^*p* < 0.05 and ^##^*p* < 0.01 compared to the MCAO group (*n* ≥ 5 each group). CTR: control; MCAO: middle cerebral artery occlusion; EA: electroacupuncture.

Apart from functional neuroplasticity biomarkers, some structural neuroplasticity biomarkers were also investigated. One-way ANOVA revealed a significant difference among the expression of the biomarker for neurons, NeuN, (*F*_2,12_ ≥ 4.283, *p* ≤ 0.0338), and synaptic plasticity-related protein PSD-95, in the hippocampus and PFC (*F*_2,12_ ≥ 6.567, *p* ≤ 0.0118) (Figure 8). The results above suggest that suppressions of the neuronal nuclear biomarker and structural synaptic protein could be found in the operation group and EA could restore these.

### 3.7. Immunofluorescence of the Synaptic Plasticity-Related Protein in the Hippocampus

**Figure 9:**
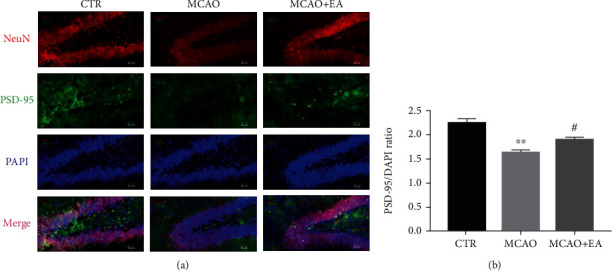
Effects of MCAO and EA on the fluorescence intensity ratio of PSD-95/DAPI in the hippocampus. The fluorescence intensity ratio of PSD-95/DAPI (b) was analyzed. Data are expressed as mean ± SEM, where ^∗∗^*p* < 0.01 compared to the control group and ^#^*p* < 0.05 compared to the MCAO group (*n* = 3 each group). CTR: control; MCAO: middle cerebral artery occlusion; EA: electroacupuncture.

In parallel to Western blotting, PSD-95 spatial information was measured in the hippocampus by immunofluorescence (Figure 9(a)). Significant treatment effects were present on the PSD-95 in the hippocampus (*F*_2,6_ = 26.98, *p* = 0.001). EA could restore the PSD-95 expression after the MCAO operation. The results are consistent with the above Western blotting results.

## 4. Discussion

In China, the prevalence of PSCI is 41.8% in ischemic stroke survivors aged ≥40 [31]. It has an adverse influence on their daily life and a growing economic burden on their family [32]. However, most current therapies, such as pharmacological treatment and cognitive training, lack of sufficient evidence [33]. Systematic reviews have proposed that acupuncture may be effective on cognitive function after stroke [34, 35]. This novel EA/TNS has applied into different kinds of cognitive impairment in clinical studies [20, 36].

In our study, the body weight of the animal was decreased after the MCAO operation, which was similar to some other studies [23, 37]. The EA treatment promoted the recovery of body weight in the I/R injury rats. This result could be mainly attributed to the multiple effects of acupuncture. In fact, similar multiple effects were observed in our clinical study [20]. EA could reduce not only cognitive deterioration of stroke patients but also some other sequelae of stroke, such as poststroke depression and functional disability [20].

According to previous studies [38–40], MCAO can induce cognitive impairment in animal behavior tests. In the current study, the rats in the MCAO group required much longer time to find the platform during the training phase and spent less time in the targeted quadrant in the probe phase, compared with the sham-operated rats. In the novel object recognition test, the MCAO group spent less time on the novel object than the control group. However, EA/TNS reversed the above phenomena in both tests. These results indicate that the MCAO group rats had not only impaired spatial learning and memory but also recognition decline, while the EA/TNS treatment could ameliorate these dysfunctions in both cognitive tests. This could provide additional animal behavior evidence to support the findings in our clinical study [20].

However, in the open field test, we found no intergroup differences in the time spent or the frequency of entry into the central zone. There was also no difference between these groups in the velocity of movement or the frequency of entry into the targeted quadrant in the water maze test, as well as the total exploration time in the novel object recognition test. These results suggest that neither the MCAO operation nor the EA treatment affected the anxiety levels or locomotor function in these three groups, which implied that the longer latency and shorter duration in the water maze test were the consequences of cognitive dysfunction, rather than of locomotor abnormality. Furthermore, these dysfunctions were the direct impact of stroke rather than anxiety.

BDNF, as a member of the neurotrophin family, is a critical molecular determinant of cell proliferation, differentiation, and synaptic modulation [41]. BDNF exerts its neuroprotective functions via binding to the TrkB receptor [42]. It can affect cognitive function via long-term potentiation (LTP), which is a powerful regulator of plasticity-related processes in long-term memory [43]. In one clinical study, BDNF levels in the serum were discovered to be much lower in patients with a history of acute ischemic stroke than in healthy individuals [44]. Likewise, in this study, we found that the BDNF level in the MCAO group was lower than that in the control group. This is also consistent with some other animal experiments [45, 46]. Meanwhile, at the downstream of the BDNF/TrkB signaling pathway, the changes in the TrkB level reflected those of BDNF in both our study and others [47, 48]. EA/TNS was found to reverse these changes in our study, suggesting that it restores cognitive function by enhancing the BDNF/TrkB pathway to contribute to neuroprotection.

Apart from its neuroprotective effects, acupuncture was also found to alter the quantity of neurotransmitter receptors; then, it might improve synaptic plasticity in this study [7]. However, overactive NMDAR [49] and AMPAR [50] can bring about excitotoxicity and neurotoxicity. Excitotoxicity appeared in the early stage of reperfusion. One study indicated that the numbers of NMDAR and AMPAR were suppressed for up to 3 days after reperfusion [51]. Nevertheless, in the long term, these receptors play decisive roles in synaptic plasticity. Our results revealed that the MCAO operation downregulated the expression of NMDAR, AMPAR, GABAAR, and CaMKII, suggesting that stroke promotes cognitive deterioration. Cotreatment with EA prevented these adverse effects of MCAO but did not induce overexpression of NMDAR or AMPAR. We could find some similar results that acupuncture had a treatment effect for neuropsychiatric disorders via modulating glutamate receptors and preventing neuronal excitotoxicity and hyperexcitability [52, 53]. Therefore, EA/TNS could restore the quantity of neurotransmitter receptors, but not overexpression.

Associated with these receptors, especially NMDAR, PSD-95 is a cytoskeletal component found at the synapses. PSD-95 enhances presynaptic neuron maturation, the quantity of postsynaptic glutamate receptors, and the quantity and size of dendritic spines [54]. It plays an essential role in synaptic plasticity and stabilization of synaptic alteration during LTP [55] and can be considered a biomarker for synaptic plasticity. In this study, the MCAO suppressed the expression of PSD-95, while EA increased it in both the PFC and hippocampus. Since spatial information cannot be ascertained from Western blotting, we used immunofluorescence to localize the PSD-95, and these two results were consistent.

EA on the trigeminal nerve-innervated acupoints is often used in the treatment of various psychiatric disorders, such as depression and obsessive-compulsive disorders, to name a few [56, 57]. These acupoints are innervated by the ophthalmic branch of the trigeminal nerve, from which the sensory information is sent to the trigeminal nucleus in the brainstem [19]. The nucleus is closely related to the dorsal raphe nucleus (DRN) containing serotonin- (5-HT-) producing neurons [58], and the locus coeruleus (LC) containing norepinephrine- (NE-) producing neurons [59], forming the brainstem reticular formation. It has additionally been proposed that DRN plays a crucial role in the brain's neural plasticity [60], neurogenesis [61], and synaptogenesis [62]. Previous studies have suggested that 5-HT could regulate BDNF expression in some stress animal models [63, 64]. The LC–NE system assumes an essential role in determining cognitive function, and NE can protect neurons from damage [65]. An in vitro study has supported that NE may be an essential modulator in BDNF expression in hippocampal neurons [66]. Our results showed that EA/TNS could enhance the BDNF/TrkB signaling pathway; this might be related to the trigeminal sensory pathway, dorsal raphe nucleus, and locus coeruleus.

**Figure 10:**
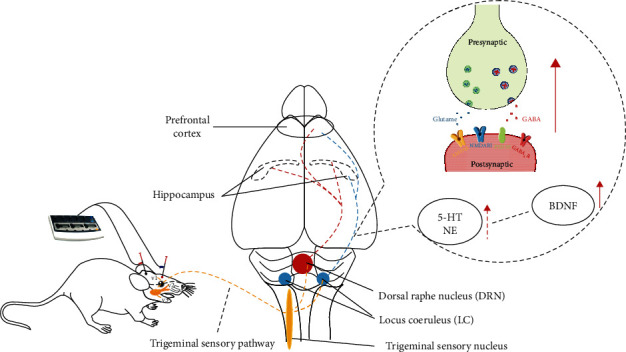
Schematic chart of the possible pathway for EA at GV20 and EX-HN3 against poststroke cognitive impairment in a rat model.

In addition to neuroprotection, BDNF could both enhance spine density in hippocampal slices in long-term stimulation [67] and promote LTP induction in short-term stimulation [68]. Apart from the structural spine plasticity, the BDNF pathway has also been shown to directly increase PSD-95 at synapses, which are mediated by the phosphatidylinositol 3-kinase signaling pathway downstream of TrkB after NNDAR activation [69]. Furthermore, BDNF is considered to play a fundamental role on neurotransmitter release [70], NMDAR transmission [71], AMPAR expression alterations [72], and GABAAR transcription [73]. Our results also revealed that EA/TNS could improve the neurotransmitter receptor quantity and synaptic plasticity-related protein; this may be related to the increased BDNF secretion. Taking the above together, we suggest that EA/TNS could induce 5-HT and NE expression in the brainstem, then enhance BDNF secretion, subsequently modulate neurotransmitter receptors and PSD-95 expression, and ultimately regulate synaptic plasticity (Figure 10).

However, some limitations of this study should be noted. First, according to a study by Yang [51], the expression of NMDAR and AMPAR may change dynamically during pathological processes. However, we only investigated the long-term (2 weeks) treatment effect. Second, we were unable to analyze the infarct volumes because the ischemic areas had severe edema, or even liquefaction, after 14 days. Third, we focused on a single protein in the postsynapses. Although this protein is related to synaptic plasticity, the evidence gathered here may be insufficient to draw definitive conclusions. Finally, the upstream of this pathway for mediation of cognitive function in the hippocampus and the PFC could be further explored and verified, especially in the brainstem.

## 5. Conclusions

To sum up, EA at *EX-HN3* (*Yintang*) and *GV20* (*Baihui*) could alleviate PSCI in the MCAO rat model. The mechanism of action appears to enhance neuroprotection and regulate synaptic plasticity in the hippocampus and PFC.

## Data Availability

The key data have been present in the context. The datasets used in this study can be obtained from the corresponding author upon reasonable request.

## Supplementary Materials

Supplementary Materials 1 This file shows that the rat could move freely in the box during the electroacupuncture treatment.
